# Pancreatic cancer induces muscle wasting by promoting the release of pancreatic adenocarcinoma upregulated factor

**DOI:** 10.1038/s12276-021-00582-2

**Published:** 2021-03-17

**Authors:** Wonbeak Yoo, Hyunji Choi, Young Hoon Son, Jaemin Lee, Seongyea Jo, Dana Jung, Yeon Jeong Kim, Sang Seok Koh, Yong Ryoul Yang, Eun-Soo Kwon, Kwang-Pyo Lee, Kyung Hee Noh, Kyung Won Kim, Yousun Ko, Eunsung Jun, Song Cheol Kim, Seokho Kim

**Affiliations:** 1 Environmental Disease Research Center, Korea Research Institute of Bioscience and Biotechnology, Daejeon, 34141 Republic of Korea; 2 Graduate School of New Drug Discovery and Development, Chungnam National University, Daejeon, 34134 Republic of Korea; 3 Aging Research Center, Korea Research Institute of Bioscience and Biotechnology, Daejeon, 34141 Republic of Korea; 4 Industrial Bio-Materials Research Center, Korea Research Institute of Bioscience and Biotechnology, Daejeon, 34141 Republic of Korea; 5 Laboratory of Stem Cells and Tissue Regeneration, Department of Biotechnology, College of Life Sciences and Biotechnology, Korea University, Seoul, 02841 South Korea; 6 Department of Health Sciences, The Graduate School of Dong-A University, Busan, 49315 Republic of Korea; 7 Department of Biological Sciences, Dong-A University, Busan, 49315 Republic of Korea; 8 Korea Research Institute of Bioscience and Biotechnology, Daejeon, 34141 Republic of Korea; 9 Department of Radiology and Research Institute of Radiology, University of Ulsan College of Medicine, Asan Medical Center, Seoul, 05505 Republic of Korea; 10 Department of Convergence Medicine, Asan Institute for Life Sciences, University of Ulsan College of Medicine and Asan Medical Center, Seoul, 05505 Republic of Korea; 11 Division of Hepato-Biliary and Pancreatic Surgery, Department of Surgery, Asan Medical Center, AMIST, University of Ulsan College of Medicine, Seoul, 05505 Republic of Korea; 12 Department of Medicinal Biotechnology, College of Health science, Dong-A University, Busan, 49315 Republic of Korea

**Keywords:** Pancreatic cancer, Pancreatic cancer

## Abstract

Cancer cachexia is a highly debilitating condition characterized by weight loss and muscle wasting that contributes significantly to the morbidity and mortality of pancreatic cancer. The factors that induce cachexia in pancreatic cancer are largely unknown. We previously showed that pancreatic adenocarcinoma upregulated factor (PAUF) secreted by pancreatic cancer cells is responsible for tumor growth and metastasis. Here, we analyzed the relation between pancreatic cancer-derived PAUF and cancer cachexia in mice and its clinical significance. Body weight loss and muscle weight loss were significantly higher in mice with Panc-1/PAUF tumors than in those with Panc-1/Mock tumors. Direct administration of rPAUF to muscle recapitulated tumor-induced atrophy, and a PAUF-neutralizing antibody abrogated tumor-induced muscle wasting in Panc-1/PAUF tumor-bearing mice. C2C12 myotubes treated with rPAUF exhibited rapid inactivation of Akt-Foxo3a signaling, resulting in Atrogin1/MAFbx upregulation, myosin heavy chain loss, and muscle atrophy. The neutrophil-to-lymphocyte ratio and body weight loss were significantly higher in pancreatic cancer patients with high PAUF expression than in those with low PAUF expression. Analysis of different pancreatic cancer datasets showed that PAUF expression was significantly higher in the pancreatic cancer group than in the nontumor group. Analysis of The Cancer Genome Atlas data found associations between high PAUF expression or a high DNA copy number and poor overall survival. Our data identified tumor-secreted circulating PAUF as a key factor of cachexia, causing muscle wasting in mice. Neutralizing PAUF may be a useful therapeutic strategy for the treatment of pancreatic cancer-induced cachexia.

## Pancreatic cancer: Protein promotes cancer-related weight loss

A protein involved in tumor growth is also a likely cause of weight loss during pancreatic cancer, and may be a useful target for prognosis and treatment. Pancreatic adenocarcinoma upregulated factor (PAUF) is secreted by pancreatic cancer cells and is also found in other cancers where significant weight loss occurs. Wonbeak Yoo, Korea Research Institute of Bioscience and Biotechnology, Daejeon, South Korea, and co-workers examined the role of PAUF in cancer-related weight loss in mice. Mice inoculated with PAUF-expressing cancer cells experienced weight loss despite no change in food consumption. PAUF increased muscle atrophy and triggered muscle wasting pathways. PAUF is routinely measured in cancer patients, and could therefore provide a convenient biomarker to inform treatment decisions. The researchers suggest that neutralising PAUF could be a valuable therapeutic option.

## Introduction

Cachexia is a metabolic syndrome characterized by decreases in skeletal muscle and fat that occurs in more than 50% of patients with advanced solid cancers^1^. The clinical features of cachexia include weight loss, inflammation, insulin resistance, and destruction of muscle protein. A reduction in skeletal muscle mass, which is a clinically important feature of cachexia, is a prognostic factor of mortality. Preventing muscle wasting thus decreases morbidity and enables continuous cancer treatment, decreasing toxicity while increasing the efficacy of chemotherapy^2–5^. Despite the severity of cancer cachexia, standardized assessment methods or established treatments remain to be identified because the etiology of this condition is unclear. The major events leading to muscle wasting in cancer-bearing hosts remain unknown.

Up to 80% of pancreatic cancer patients develop cachexia during the disease course^6^. One-third of pancreatic cancer patients die from complications of cachexia, including severe respiratory muscle damage because of cardiopulmonary dysfunction and immune dysfunction^7^. More than 70% of newly diagnosed pancreatic cancer patients meet cachexia criteria, and although nearly 70% of pancreatic cancers are diagnosed as unresectable, pancreatic cancer patients who undergo resection show cachexia, and 40% of patients eligible for resection show weight loss^8^. Even in patients who undergo resection, cachexia is associated with poor responses to treatment and decreased survival rates. In patients receiving FOLFIRINOX chemotherapy, a reduction in adipose tissue and muscle atrophy are critical factors associated with pancreatic ductal adenocarcinoma mortality. The identification of a cancer cachexia-associated biomarker would be valuable to improve the outcomes of pancreatic cancer patients.

Numerous soluble factors, including systemic hormones and inflammatory cytokines, are considered cancer cachexia biomarkers associated with systemic inflammation^9–11^. However, inflammatory cytokines associated with cachexia, including tumor necrosis factor (TNF), interleukin (IL)-6, IL-1β, and interferon (IFN)-γ, are unlikely to be considered potential biomarkers associated with cachexia. This is because inflammatory cytokines associated with disease progression are difficult to distinguish from those secreted in association with second-line or third-line chemotherapy. In addition, the severity of muscle wasting in pancreatic cancer cannot be predicted by only the tumor burden. Resectable pancreatic cancer patients have low levels of canonical cytokines, including TNFα, IL-6, IL-1β, and IFN-γ, which are not correlated with cancer cachexia. However, serum monocyte chemoattractant protein-1 levels in resectable pancreatic cancer patients are highly correlated with cancer cachexia^8^. Therefore, distinguishing tumor-specific and host-specific factors that affect cancer-induced cachexia may be essential for screening therapeutic targets and identifying the patient population that may benefit from targeted therapies.

Pancreatic adenocarcinoma upregulated factor (PAUF), also known as ZG16b, has been extensively investigated as a tumorigenic factor and diagnostic marker in pancreatic cancer as well as gastric, colon, ovarian, oral squamous cell, and cervical carcinoma^12,13^. PAUF promotes angiogenesis and recruits myeloid-derived suppressor cells, which mediate immune evasion in the tumor microenvironment. PAUF facilitates tumor growth and plays a central role in stromal cells present in the tumor microenvironment by affecting autocrine/paracrine signaling. In a previous study, we showed that mice injected with PAUF-overexpressing cells had poor mortality rates, even those with tumors of the same size^14,15^. These data suggest that PAUF plays a role in cachexia-induced morbidity during pancreatic cancer progression. In the present study, we investigated the effect of PAUF secretion by pancreatic cancer cells on cachexia induction in an in vivo mouse model of PAUF-modified tumors. To generate the in vivo model, PAUF-overexpressing pancreatic cancer cells were implanted into mice. In addition, recombinant PAUF (rPAUF) was injected directly into muscle tissues. Mice with PAUF-overexpressing tumors were treated with a PAUF-neutralizing antibody to examine the relationship between PAUF and cachexia. Finally, we showed that the levels of PAUF were increased in the plasma of patients in correlation with cancer-induced cachexia phenotypes. The present findings demonstrate that PAUF secreted by pancreatic cancer cells induces muscle atrophy in an Atrogin-1-dependent manner in pancreatic cancer.

## Materials and methods

### Cell culture

The Panc-1 human pancreatic cancer cell line and C2C12 murine myoblast cell line were obtained from the American Type Culture Collection (ATCC; Manassas, VA, USA) and were maintained in ATCC-recommended growth medium at 37 °C and 5% CO_2_. The generation of PAUF-overexpressing (Panc-1/PAUF) cell lines and controls was described in a previous report^14^. Panc-1/Mock, Panc-1/PAUF, and C2C12 cells were cultured in Dulbecco’s modified Eagle’s medium (DMEM) supplemented with 10% fetal bovine serum (FBS) and 1% antibiotics. To induce myogenic differentiation, C2C12 myoblasts were grown to 80% confluence (day 0) and cultured in DMEM containing 2% FBS and 1% antibiotics. Conditioned media were collected from Panc-1/Mock and Panc-1/PAUF cell cultures after 24 h, pooled, and sterilized by filtration through a 0.22-μm filter. To analyze the effect of PAUF on muscle atrophy, C2C12 cells were differentiated for 5 days and then treated with conditioned medium collected from control (Panc-1/Mock) or PAUF (Panc-1/PAUF) cultures for 24 h. To analyze myotube diameters, cells were fixed with 10% formalin, washed in phosphate-buffered saline (PBS), and stained with eosin. The diameters were calculated using NIH ImageJ software (http://rsb.info.nih.gov/ij).

### PAUF ELISA

Cultured supernatants from Panc-1/Mock or Panc-1/PAUF cells and plasma from pancreatic cancer patients were used for the detection of PAUF using the methods described by Choi et al.^12^. Plates were coated with 4E6 capture antibodies (5 μg/ml) for 16 h at room temperature and incubated with patient-derived plasma for 2 h. Detection antibodies (11G6, 250 ng/ml) were added for 90 min at 37 °C, followed by incubation with streptavidin-HRP (1:5000) for 30 min at 37 °C. The PAUF expression level was detected at 450 nm.

### Animal experiments

All animal studies were performed in compliance with the policies of the Korea Research Institute of Bioscience and Biotechnology (KRIBB Daejeon, Korea) Animal Care and Use Committee (approval number: KRIBB-ACE-18162). Mice were maintained on a 12-h light/12-h dark cycle with food and water provided ad libitum. NOD-scid gamma (NSG) mice (The Jackson Laboratory, Bar Harbor, ME, USA) were used in all experiments and housed in a pathogen-free animal facility at KRIBB. To establish the pancreatic cancer cachexia model, 5.0 × 10^5^ Panc-1/Mock or Panc-1/PAUF cells were slowly injected into the pancreas tail of mice (6 weeks old) under anesthesia (isoflurane, Butler Schein, Dublin, OH, USA). To assess muscle wasting induced by PAUF, mice were treated with PBS or 50 μg/kg rPAUF by intraperitoneal injection every 2 days for 3 weeks or with intramuscular injection of Matrigel (Becton Dickinson, Oxford, Oxon, UK) containing 50 μg/kg rPAUF. We chose 50 μg/kg rPAUF for this study based on the optimal dose in experiments with tumor-derived factors^16,17^. For PMAb83 experiments, mice were treated with 10 mg/kg control IgG or PMAb83 starting at 1 week postimplantation twice a week for 3 weeks. rPAUF and PMAb83 were generated as previously described^18,19^. Body weight and food intake were assessed weekly using scales. More than ten mice per group were used in experiments, and duplicates or triplicates were performed in each experiment.

### Immunohistochemical analysis

Harvested mouse tissues from the liver, adipose tissue, or skeletal muscle were fixed in 10% formalin and then mounted in OCT for cryosectioning or paraffin for immunohistochemical staining. The frozen samples were cryosectioned at a thickness of 10 μm. Sections were then blocked in 1% BSA in PBS for 1 h and incubated with an anti-laminin antibody (Abcam, Cambridge, MA, USA) overnight at 4 °C. The Alexa Fluor 488 (Abcam, Cambridge, MA, USA) fluorescent dye conjugated to a secondary antibody and DAPI (Vector Laboratories, Burlingame, CA) were used for visualization. The paraffin sections were stained with hematoxylin for 30 s and then with eosin for 60 s. Measurements were performed using NIH ImageJ software (http://rsb.info.nih.gov/ij).

### Western blot analysis

Cells were rinsed in PBS and lysed in Pro-Prep lysis buffer (iNtRON Biotechnology, Seoul, South Korea) containing protease and phosphatase inhibitors (Sigma Aldrich, St. Louis, MO, USA). The protein concentration was determined using a bicinchoninic acid (BCA) protein assay (Pierce Biotechnology, Rockford, IL, USA). Individual proteins were separated by SDS-PAGE and transferred to PVDF membranes (Millipore, Billerica, MA, USA). The membranes were probed with the appropriate primary antibodies diluted in PBS with Tween-20 containing 1% BSA and then with secondary horseradish peroxidase-conjugated antibodies. Bound antibodies were visualized using ECL reagents (Millipore). The following primary antibodies were used for western blotting: anti-IRα, anti-IRS-1, anti-p-mTOR, anti-mTOR, anti-pS6K, anti-S6K, anti-pAkt, anti-Akt, anti-p-ERK, anti-ERK, anti-Foxo3a, anti-pFoxo3a, and anti-glyceraldehyde-3-phosphate dehydrogenase (GAPDH) antibodies from Cell Signaling Technology (Denver, MA, USA); anti-Atrogin-1 and anti-MuRF-1 antibodies from ECM Biosciences (Versailles, KY, USA); an anti-ubiquitin antibody from AbFrontier (Seoul, Korea); and an anti-α-tubulin antibody from Sigma-Aldrich (St. Louis, MO, USA).

### Data collection from patients with pancreatic cancer

Between December 2017 and May 2018, peripheral blood was drawn from 20 patients with pancreatic cancer who were diagnosed by pathological testing at the Asan Medical Center, a tertiary care center. The plasma was separated and stored at −70 °C until analysis. Patients who did not consent and those whose condition could interfere with the study results (e.g., infectious diseases or coexisting illness) were excluded. The study complied with the Declaration of Helsinki, and the protocol was reviewed and approved by the Institutional Review Board of the Asan Medical Center (IRB No. 2017–0305). Written informed consent was obtained from all participants. Patient-, surgery-, and oncology-related data were obtained from patient medical records. Patient body weight before tumor diagnosis was obtained from the patient’s past history, which was usually recorded 6 months to 1 year before tumor diagnosis. We obtained blood samples when patients were hospitalized for cancer evaluation. Only two of the enrolled patients were treated with preoperative chemotherapy, and the other patients had no additional treatment, such as radiation therapy. Data are shown as the mean ± standard deviation. For statistical analysis of the data, patients were divided into two groups on the basis of plasma PAUF levels as follows: a low-PAUF group (<10.2 ng/ml) and high-PAUF group (≥10.2 ng/ml).

### Assessment of skeletal muscle and fat areas

Body composition, i.e., the skeletal muscle area (SMA) and intermuscular fat area (IMA), was assessed at the L3 vertebral level on an abdominopelvic CT scan. An experienced radiologist (K.W.K., 13 years of experience) analyzed the CT images using automated AsanJ-Morphometry^TM^ software (AsanJ-Morphometry, available online: http://datasharing.aim-aicro.com/morphometry, accessed on 17 October 2019). The SMA and IMA were demarcated using predetermined thresholds (−29 to +190 and −190 to −30 Hounsfield units [HU], respectively)^20^. The skeletal muscle index (SMI) was calculated by dividing the SMA by the patient body weight (cm^2^/kg)^21^. The myosteatosis index (MI) was also calculated by dividing the IMA by the patient body weight (cm^2^/kg).

### Pathway network identification and bioinformatic analyses

The interaction pairs of pathway-enriched genes were researched through the Search Tool for the Retrieval of Interacting Genes (STRING) database, version 10.5 (http://string-db.org). Known and predicted associations were integrated and scored. The pancreatic cancer patient datasets used in this study included GSE15471, GSE16515, GSE28735, GSE32676, GSE55643, GSE62165, and the TCGA. Survival analysis was performed using PROGgeneV2 (watson.compbio.iupui.edu/chirayu/proggene/database), and correlation analysis between gene copy number and survival probability was performed using the UCSC Xena database (http://xena.ucsc.edu/).

### Statistical analysis

Data were obtained from at least three experiments performed in triplicate and analyzed using Student’s *t*-test. For calculation of two-tailed significance levels for equality of means, equal variance was assumed for the two populations analyzed. The relations between variables were assessed by using Pearson’s regression analyses. The chi-squared test or Mann–Whitney *U*-test was used to compare patient characteristics. All statistical analyses were performed using GraphPad PRISM 5 (GraphPad, San Diego, CA, USA). Results were considered significant when *p*-values were <0.05.

## Results

### PAUF induces cancer cachexia in mice with pancreatic tumors

**Fig. 1:**
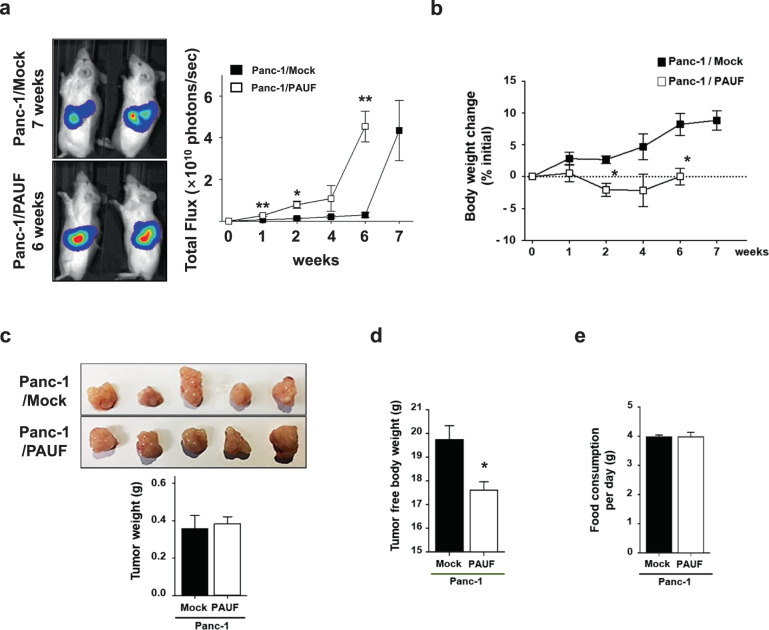
Association between cachexia and pancreatic cancer-derived PAUF. **a** Panc-1/Mock or Panc-1/PAUF cells were orthotopically injected into NSG mice (*n* = 5). Bioluminescence imaging of luciferase in the Panc-1/Mock or Panc-1/PAUF tumor-bearing mice and photon values of the tumor mass are displayed in the graph. **b** Time-dependent changes are expressed as the percent modulation of initial body weight. **c** Representative tumor image and tumor weight after Panc-1/Mock (7 weeks) or Panc-1/PAUF (6 weeks) cell inoculation. **d** Tumor-free body weight. **e** Food consumption per day. The data represent the mean ± SD; *p < 0.05 vs. Panc-1/Mock cell-injected mice.

To examine the effect of PAUF on cancer cachexia, the Panc-1 cell line, which had no expression of PAUF among the pancreatic cancer cell lines tested^18^, was chosen as an adequate in vivo tumor model. PAUF-overexpressing Panc-1 or control cells with stable luciferase expression (Panc-1/PAUF-Luc or Panc-1/Mock-Luc) were generated, tested for the expression and secretion of PAUF (Supplementary Materials, Fig. s1), and then orthotopically injected into NSG mice. As expected, implanted Panc-1/PAUF tumors grew significantly faster than Panc-1/Mock tumors (Supplementary Materials, Fig. s2a). Interestingly, mice inoculated with Panc-1/PAUF cells showed a decrease in total body weight without changes in food consumption (Supplementary Materials, Fig. s2b, c). At the end of the experiment, tumors were removed from tumor-bearing mice, and the weight of the tumor-free bodies was measured. Consistently, tumor weight was higher in the Panc-1/PAUF group than in the Panc-1/Mock group (Supplementary Materials, Fig. s2d), and the tumor-free body weight of the Panc-1/PAUF-injected group was lower than that of the control group (Supplementary Materials, Fig. s2e). White adipose and liver tissues were not significantly different between the two groups, while the weight of tibialis anterior (TA) muscles was significantly decreased in the Panc-1/PAUF group (Supplementary Materials, Fig. s2f). The amount of body weight loss can positively correlate with tumor volume^22^. Because overexpression of PAUF was shown to promote tumor progression (Supplementary Materials, Fig. s2), experiments were designed to consider tumor volume. Moreover, when Panc-1/Mock or Panc-1/PAUF tumors reached similar sizes by IVIS imaging of the luciferase signal, the mice were necropsied at different time points (Fig. 1a). As shown Fig. 1b, Panc-1/PAUF tumor-bearing mice showed a decrease in total body weight, and these results were in agreement with the aforementioned data (Supplementary Materials, Fig. s1). Interestingly, tumor weights were not changed (Fig. 1c), while tumor-free body weights were consistently decreased in the Panc-1/PAUF group compared with the Panc-1/Mock group (Fig. 1d). However, food consumption was not different between the two groups (Fig. 1e). Because the Panc-1 cell line does not express PAUF, pancreatic cancer cells with high expression of PAUF were chosen, and body weight changes in a tumor-bearing mouse model were evaluated. Consistent with our observations above (Supplementary Materials, Fig. s1 and Fig. 1), subcutaneous inoculation of mice with a high intrinsic PAUF-expressing cell line, such as MIA PaCa-2 or CFPAC-1, resulted in body weight loss without changes in food consumption compared to subcutaneous inoculation of control cells (Supplementary Materials, Fig. s3a–d). These results indicate that tumor formation by PAUF-expressing cells results in overall body weight loss because of cachexia in mice.

### PAUF is associated with increased muscle atrophy

**Fig. 2:**
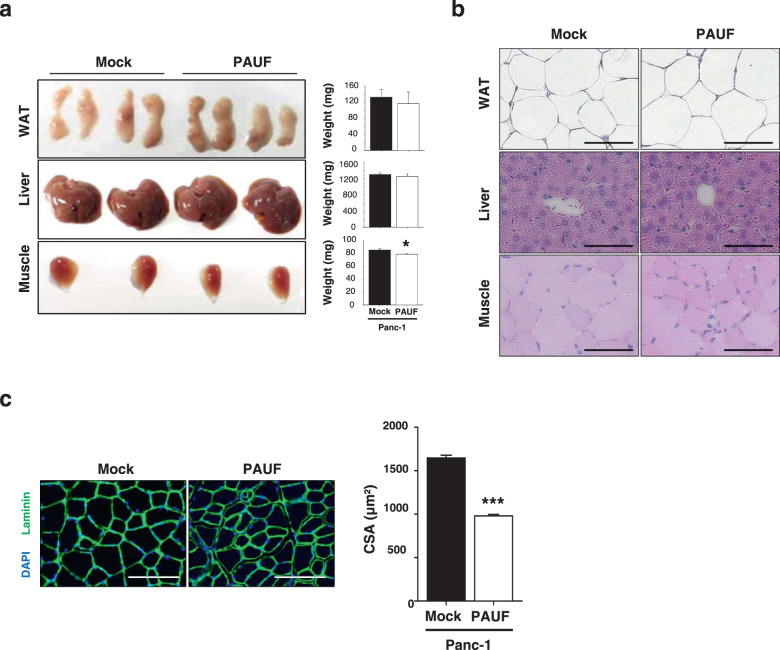
Tissue histology of Panc-1/Mock and Panc-1/PAUF tumor-bearing mice. **a** Representative images of white adipose tissue (WAT), liver tissue, and tibialis anterior (TA) muscles from Panc-1/Mock or Panc-1/PAUF tumor-bearing mice are shown. Weights of WAT, liver tissue, and the TA muscle. **b** Representative images of hematoxylin and eosin staining of WAT, liver tissue, and the TA muscle. **c** Representative images of muscle sections stained for laminin. Scale bar, 100 µm. The graph shows the mean CSA of muscle myofibers. The data represent the mean ± SD; *p < 0.05, **p < 0.01, and ***p < 0.001 vs. Panc-1/Mock cell-injected mice.

To determine whether the PAUF-induced decrease in body weight was caused by decreases in the weights of multiple tissues/organs, mice were sacrificed, and the weights of several organs were measured. The weight of the TA muscle differed significantly between Panc-1/PAUF and control mice (Fig. 2a), while there were no significant change in heart, white adipose tissue (WAT), or liver mass in response to tumor-released PAUF (Fig. 2b). TA muscle sections were stained with H&E and an anti-laminin antibody to examine the effect of PAUF on muscle fiber architecture and cross-sectional area (CSA). Laminin staining showed that muscle fiber size was smaller in Panc-1/PAUF-inoculated mice than in control mice (Fig. 2c). Similarly, the mice bearing tumors generated by MIA PaCa-2 or FAPAC-1 cells, which express high intrinsic levels of PAUF, revealed smaller muscle fibers than control mice (Supplementary Materials, Fig. s3e). These results indicate that PAUF released from cancer cells induces muscle atrophy leading to body weight loss in pancreatic cancer models.

### rPAUF induces body weight loss

**Fig. 3:**
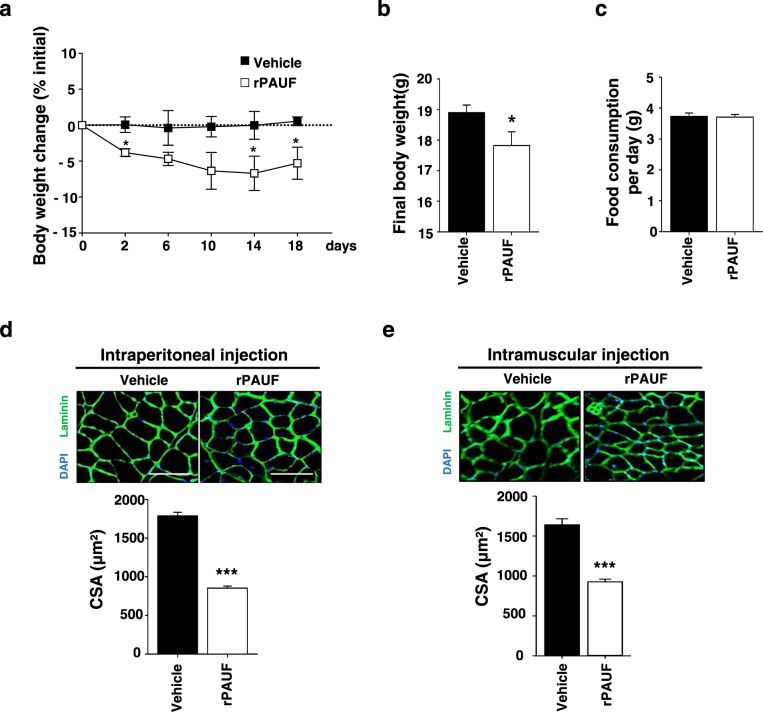
rPAUF induces body weight loss and muscle atrophy. **a** Time-dependent changes expressed as the percent modulation of initial body weight in mice injected intravenously with rPAUF (50 µg/kg) or PBS. **b** Final body weight at 18 days postinjection. **c** Food consumption per day. **d** Representative images of muscle sections stained for laminin. Scale bar, 100 µm. The graph shows the mean CSA of muscle myofibers. **e** rPAUF (50 µg/kg) or PBS was injected into the muscles of mice. Representative images of muscle sections stained for laminin are shown. Scale bar, 100 µm. The graph shows the mean CSA of muscle myofibers. The data represent the mean ± SD; *p < 0.05, **p < 0.01, and ***p < 0.001 vs. PBS.

To determine whether PAUF has a direct effect on muscle wasting in the absence of tumors, mice were injected intraperitoneally with rPAUF, which was produced in a CHO cell system, every 2 days for 2 weeks, and body weight changes were monitored. Mice began to lose body weight 2 days after injection of rPAUF. By 18 days postinjection of rPAUF, mice had lost 8% of their starting body weight. On day 2, mice injected with rPAUF showed an approximately 4% loss of body weight, which progressed to 8% after 18 days (Fig. 3a, b). Food consumption did not differ significantly between the two groups (Fig. 3c). At the end of the experiment, TA muscles were excised and stained with DAPI and an anti-laminin antibody to examine the effect of rPAUF on muscle fiber architecture and CSA. Immunofluorescence data revealed that the CSA was lower in rPAUF-injected mice than in control mice, and similar results were obtained in mice inoculated with PAUF-expressing cancer cells (Fig. 3d). To examine the role of rPAUF in muscle fiber atrophy, we directly injected Matrigel mixed with rPAUF into the TA muscle. After 2 weeks, immunofluorescence analysis showed that the size of muscle fibers was smaller in rPAUF-injected mice than in control mice (Fig. 3e). Taken together, these results indicate that PAUF induced skeletal muscle catabolism independently of cancer cells.

### PMAb83, a PAUF-neutralizing antibody, attenuates PAUF-induced muscle atrophy in pancreatic cancer models

**Fig. 4:**
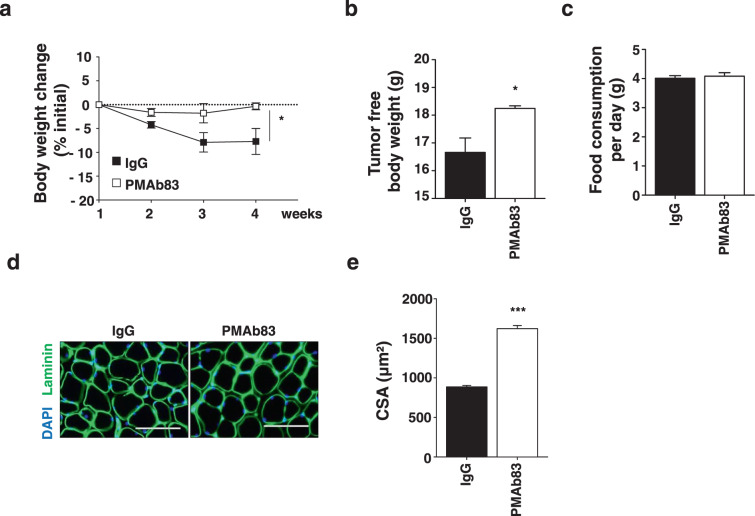
PMAb83 reduces muscle atrophy in PAUF tumor-bearing mice. **a** Panc-1/PAUF cells were orthotopically injected into NSG mice. Two weeks after tumor cell injection, the tumor-bearing mice were treated with control IgG (10 mg/kg) or PMAb83 (10 mg/kg) intraperitoneally twice a week. Time-dependent changes are expressed as the percent modulation of initial body weight in the ctrl IgG- or PMAb83-injected mice. **b** Tumor-free body weight. **c** Food consumption per day. **d** Representative images of muscle sections stained for laminin. Scale bar, 100 µm. **e** The graph shows the mean CSA of muscle myofibers. The data represent the mean ± SD; *p < 0.05, **p < 0.01, and ***p < 0.001 vs. ctrl IgG.

PMAb83 is a PAUF-neutralizing antibody that was developed as a targeted agent for the treatment of pancreatic cancer. To confirm the effect of PMAb83 on cachexia, Panc-1/PAUF cells were implanted into the pancreas of NSG mice, which were then treated with PMAb83 (10 mg/kg) twice a week starting at 5 days after tumor cell injection. At 4 weeks after administration of IgG or PMAb83, body weights were not significantly changed in PMAb83-injected mice, while IgG-injected mice showed weight loss (Fig. 4a). Interestingly, tumor-free body weight was higher in PMAb83-injected mice than in IgG-injected mice (Fig. 4b). Food consumption did not differ between the two groups (Fig. 4c). Next, TA muscle were excised from mice in the two groups and stained with DAPI and an anti-laminin antibody to calculate the CSA. Consistently, the CSA was significantly higher in PMAb83-injected mice than in control mice (Fig. 4d). These results demonstrate that PMAb83 decreased PAUF-induced muscle atrophy in vivo (Fig. 4e).

### PAUF activates a catabolic pathway by inhibiting anabolic signaling in muscles

**Fig. 5:**
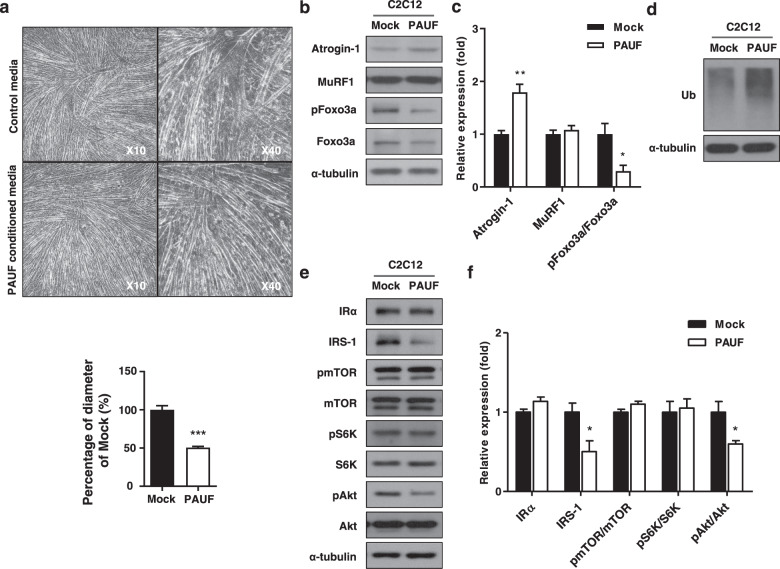
Myotube atrophy induced by PAUF conditioned medium. **a** Light microscopy images (left: low magnification at 10×; right: high magnification at 40×) showing the effect of control medium (from Panc-1/Mock cell cultures) or PAUF conditioned medium (from Panc-1/PAUF cell cultures). Relative diameter of myotubes cultured in control or PAUF conditioned medium. Four different views were randomly selected for diameter measurements and quantified using ImageJ software. Data were normalized to the diameter of myotubes cultured in control medium. **b** Western blot analysis of catabolic markers in cells treated with rPAUF using antibodies against Atrgoin-1, MuRF1, pFoxo3a, and Foxo3a. **c** Relative expression was measured by densitometric analysis of western blot data (*n* = 3). The data represent the mean ± SD; *p < 0.05 and **p < 0.01. **d** Whole-cell extracts were also subjected to SDS-PAGE followed by western blot analysis using an anti-ubiquitin antibody to assay steady-state ubiquitination levels. **e** Western blot analysis of anabolic markers in cells treated with rPAUF using antibodies against IR, IRS-1, pmTOR, mTOR, pS6K, S6K, pAkt, and aAkt. **f** Relative expression was measured by densitometric analysis of western blot data (*n* = 3). The data represent the mean ± SD; *p < 0.05.

To identify the molecules involved in PAUF-induced muscle atrophy in vitro, we used C2C12 murine skeletal myoblast models of atrophy. Differentiated C2C12 myotubes were exposed to PAUF conditioned medium, and myotube diameter was measured. The results showed that the diameter of PAUF conditioned medium-treated myotubes was approximately 50% smaller than that of control myotubes (Fig. 5a). Atrogin-1 and MuRF-1 are ubiquitin ligases that induce skeletal muscle atrophy by targeting proteins for degradation^23–25^. We therefore examined Atrogin-1 and MuRF-1 levels in atrophic C2C12 myotubes by western blotting. The results showed that PAUF conditioned medium upregulated Atrogin-1 by approximately 1.7-fold compared with control medium, whereas the levels of MuRF-1 were not affected by Panc-1/PAUF conditioned medium (Fig. 5b, c). Next, we analyzed Foxo3a phosphorylation, which leads to Foxo3a translocation into the cytoplasm and downregulation of Atrogin-1, in C2C12 cells treated with conditioned medium by western blotting. As shown Fig. 5b, c, the levels of phospho-Foxo3a were lower in Panc-1/PAUF conditioned medium-treated myotubes than in control myotubes. Therefore, the levels of ubiquitination were higher in the PAUF conditioned medium-treated cells than in the control cells (Fig. 5d). In general, it has been established that muscle atrophy induces an imbalance between anabolic and catabolic pathways^26,27^. Therefore, we observed the expression and activity of protein synthesis markers representing anabolism. Interestingly, myotubes treated with conditioned medium showed reductions in IRS-1 and phospho-Akt (Fig. 5e, f). Taken together, these results suggest that PAUF-induced muscle atrophy is associated with Atrogin-1-dependent protein ubiquitination in myotubes.

### PAUF is associated with cancer cachexia in patients with pancreatic cancer

**Fig. 6:**
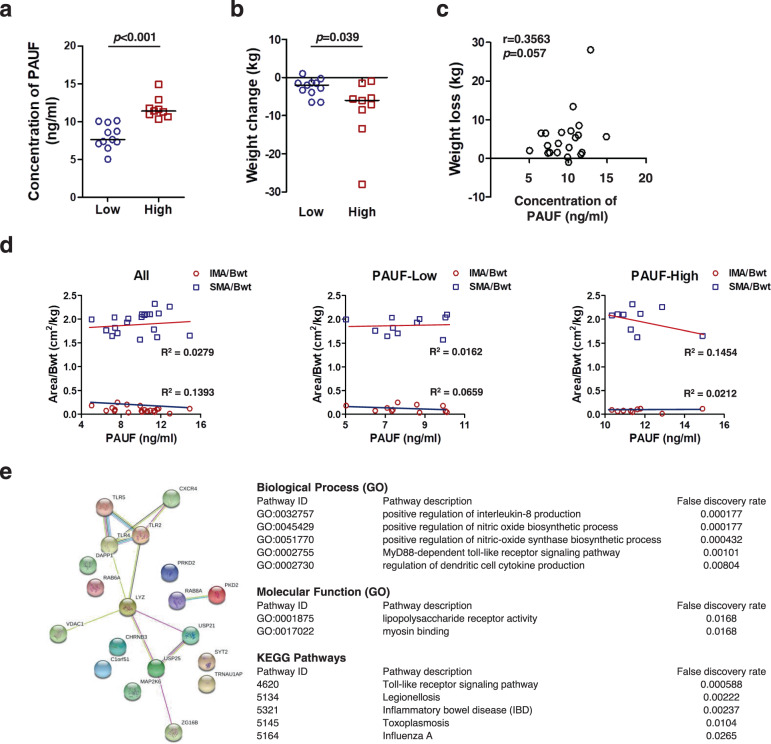
Plasma PAUF levels in pancreatic cancer patients and PAUF-associated gene analysis. **a** Plasma PAUF levels and **b** body weight changes in pancreatic cancer patients; horizontal lines indicate the median. **c** Pearson’s correlation analysis of the level of plasma PAUF and body weight loss in patients with pancreatic cancer. **d** Analysis of the skeletal muscle area and intermuscular fat area determined by abdominal CT scans in patients (SMA skeletal muscle area, IMA intermuscular fat area). **e** Graphs represent the number of associated genes (STRING [Search Tool for the Retrieval of Interacting Genes], version 10.5) for PAUF. The tables represent the significant biological processes, molecular functions, cellular components, and key pathways evaluated using STRING.

To examine whether plasma PAUF concentrations are related to disease severity, we focused on a cohort of patients with pancreatic cancer. For this analysis, we divided the pancreatic cancer patients into two groups based on the plasma PAUF concentration: <10.2 ng/ml and ≥10.2 ng/ml. The baseline characteristics of the patients with pancreatic cancer are summarized in Table 1 and Supplementary Materials, Table s1. In the 20 patients included in the analysis, the subjects with a high PAUF level were significantly associated with weight loss greater than 5% of body weight, which is defined as a cachexia indicator (*p* = 0.032). In addition, the percentages of eosinophils (3.7 ± 2.9% vs. 2 ± 2.7%; *p* = 0.027) and lymphocytes (32.9 ± 8.2% vs. 24.4 ± 6.3%; *p* = 0.023) were significantly lower in the high-PAUF group than in the low-PAUF group. However, the percentage of neutrophils, which play a role in the induction of cancer cachexia^28–30^, was higher in the high-PAUF group than in the low-PAUF group (64.5 ± 7.3% vs. 56.3 ± 8.7%; *p* = 0.058). The neutrophil-to-lymphocyte ratio (NLR), an inflammatory index^29^, was significantly higher in the high-PAUF group than in the low-PAUF group (2.9 ± 1.1 vs. 1.9 ± 0.8; *p* = 0.023). The patients in the high-PAUF group were significantly younger than those in the low-PAUF group (68.2 ± 6.7 years vs. 57 ± 10.6 years; *p* = 0.026). There were no differences in sex, BMI, food intake, body weight, WBC counts, C-reactive protein, albumin, or tumor stage between the two groups. Assessment of PAUF levels in the plasma of patients showed significant differences between the two groups (Fig. 6a). Body weight loss was significantly higher in the high-PAUF group than in the low-PAUF group (−8.5 ± 8.2 kg vs. −2.6 ± 2.3 kg; *p* = 0.039) (Fig. 6b). Next, clinical significance was analyzed using Pearson’s correlation analysis to determine the relationship between plasma PAUF and weight loss and a positive correlation was found (*r* = 0.3563, *p* = 0.057) between plasma PAUF and weight loss (Fig. 6c). Through CT imaging at the time of tumor diagnosis, the SMA and IMA of the patients were analyzed (Fig. 6d and Supplementary Materials, Fig. s4). Comparison of the PAUF-Low group and the PAUF-High group showed that patient muscle volume decreased as the PAUF level increased. To obtain a more concise understanding of the interactions between enriched pathways related to PAUF, PAUF protein–protein interaction pathways were analyzed using STRING (Fig. 6d). Biological and Kyoto Encyclopedia of Genes and Genomes (KEGG) pathway analyses showed that PAUF is associated with IL-8, nitric oxide biosynthesis, Toll-like receptor (TLR) signaling, and TNF-α processes, which are responsible for inflammatory and autoimmune diseases. Additionally, PAUF is associated with lipopolysaccharide receptor activity, which is involved in inflammation and myosin binding according to molecular function analysis.

**Table 1:** Clinical and histopathological characteristics of pancreatic cancer patients.

Variable	ALL	Low PAUF (PAUF < 10.2 ng/ml)	High PAUF (PAUF ≥ 10.2 ng/ml)	*p*-Value
Age	63.7 (±9.9)	68.2 (±6.7)	57 (±10.6)	**0.026**^a^
Sex: *n*, %				0.064^b^
Male	11 (55)	4 (57.1)	7 (42.9)	
Female	9 (45)	7 (77.8)	2 (22.2)	
BMI, kg/m^2^	24.7 (±6.8)	25 (±6.9)	24.4 (±7.1)	0.879^a^
Food intake, kcal/kg/day	26.4 (±7.7)	27.1 (±7.8)	26.3 (±8.0)	1.000^a^
Body weight, kg				
Usual weight	67.8 (±10.9)	64.3 (±9.5)	73 (±11.1)	0.087^a^
Preoperation day	63 (±9.0)	61.7 (±9.3)	64.4 (±8.8)	0.704^a^
Weight changes: *n*, %				**0.032**^**b**^
<5%	9 (45)	7 (63.6)	2 (22.2)	
≥5%	11 (55)	4 (36.4)	7 (77.8)	
Tumor stage, TNM				0.873^b^
I–II	14 (70)	8 (57.1)	6 (42.9)	
III–IV	6 (30)	3 (50)	3 (50)	
WBC, ×10^3^/μL	6.8 (±1.7)	6.9 (±1.7)	6.6 (±1.7)	0.704^a^
Lymphocyte, %	29.1 (±8.4)	32.9 (±8.2)	24.4 (±6.3)	**0.023**^**a**^
Neutrophil, %	60 (±8.9)	56.3 (±8.7)	64.5 (±7.3)	**0.058**^a^
Eosinophil, %	2.9 (±2.9)	3.7 (±2.9)	2 (±2.7)	**0.027**^**a**^
Neutrophil-to-lymphocyte ratio		1.9 (0.8)	2.9 (1.1)	**0.023**^**a**^
C-reactive protein, mg/dL	0.5 (±0.6)	0.5 (±0.7)	0.4 (±0.4)	1.000^a^
Albumin, g/dL	3.6 (±0.5)	3.7 (±0.3)	3.5 (±0.6)	0.849^a^

Values are the means ± standard deviations.

*BMI* body mass index, *WBC* white blood cell.

^a^Mann–Whitney *U*-test.

^b^Chi-squared test.

Bold values indicate statical significance *P* < 0.05.

### PAUF overexpression is associated with poor clinical outcomes in pancreatic cancer patients

**Fig. 7:**
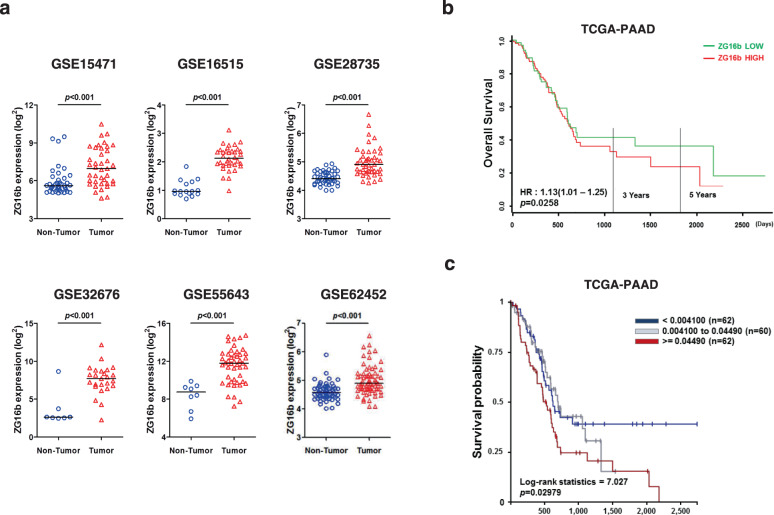
Aberrant expression of PAUF (ZG16b) and Kaplan–Meier-analyzed overall survival in pancreatic cancer. **a** PAUF expression in nontumor and tumor groups determined from gene expression data from NCBI GEO (accession numbers GSE15471, GSE16515, GSE28735, GSE32676, GSE55643, and GSE62452). **b** Relationship between PAUF expression and overall survival in the TCGA pancreatic cancer cohort (TCGA-PAAD). Gene expression was dichotomized into high and low values using the mean as the cutoff. HR, hazard ratio. **c** Kaplan–Meier survival analysis and the log-rank test were used to analyze the overall survival of patients according to the copy number of the PAUF gene in TCGA-PAAD.

The present data suggest that high PAUF expression is involved in cachexia development in pancreatic cancer patients. In addition, we previously reported aberrant expression of PAUF in pancreatic cancer cells^31^. To generalize our findings, we recapitulated PAUF gene expression from large cohorts of pancreatic cancer patients available from the GEO database. As shown in Fig. 7a, PAUF gene expression was significantly increased in the tumor groups in all the cohorts. Finally, we investigated the potential correlation of PAUF with patient survival. Log-rank analysis showed that groups with high expression of PAUF had shorter survival times than those with low expression (*p* = 0.0258; hazard ratio = 1.13, Fig. 7b). To determine the prognostic implications of copy number gains, survival analysis was performed with TCGA-PAAD. Kaplan–Meier survival analysis showed that a high copy number of the PAUF gene was correlated with a poor prognosis (*p* = 0.02979; log-rank statistics = 7.027, Fig. 7c). Analysis of multiple cohorts of pancreatic cancer patients profiled across various microarray platforms identified a strong association between PAUF and poor clinical outcomes in pancreatic cancer patients.

## Discussion

Cachexia is particularly prominent in patients with gastric, pancreatic, prostate, colon, or lung cancer^32–34^. Cachexia impairs quality of life, reduces weight, causes muscle wasting, impairs the response to chemotherapy and is a prognostic factor for the survival of cancer patients^5,35–40^. Cancer cachexia is a systemic disease that involves tumor-derived mediators, hormones, neuropeptides, abnormal host metabolism, and proinflammatory cytokine networks affected during cancer progression^32,33,36^. This severe disease is common in many types of cancer, although it is particularly ubiquitous in pancreatic cancer. There are no effective medical treatments for cancer-associated cachexia and no approved drug therapies. Here, we demonstrated that PAUF directly mediates cancer-induced muscle wasting. Previous reports show that PAUF plays important roles in the metastasis, angiogenesis, and progression of pancreatic cancer^15,18,41^. In this study, we provide novel insight into the physiological roles of PAUF in cancer-induced muscle wasting. PMAb83 suppressed cancer-related muscle wasting induced by PAUF. The functional mechanism was PAUF leading to muscle wasting through a Foxo3a-dependent pathway. These data suggest that PAUF is a key regulator causing muscle wasting in pancreatic cancer.

PAUF is a tumorigenic protein involved in pancreatic cancer^18,42^, and it is overexpressed in many human cancers in association with cachexia (Supplementary Materials, Fig. s5). In a previous study from our group, we showed that PAUF binds to TLRs 2 and 4, which upregulates AP-1-regulated genes via the major mitogen-activated protein kinase (MAPK) pathway and promotes escape from innate immune surveillance and tumor growth^43^. Consistent with our study, a recent report showed that MyD88, the universal adaptor protein for TLRs, promotes tumor progression, worsens cachexia, and accelerates mortality in vivo^44^. These results are consistent with those of the present study, which demonstrate the role of PAUF in the physiological and pathophysiological processes associated with the development of pancreatic cancer cachexia. PAUF increased catabolism by downregulating essential protein synthesis signaling, such as that by IRS-1 and phospho Akt. Inhibition of the protein synthesis pathway triggers protein degradation^45^. In addition, decreased Akt pathway activity induces Atrogin-1 expression through Foxo signaling^46^. PAUF activated catabolism by upregulating the muscle-specific E3 ligase Atrogin-1/MAFbx and triggering protein degradation through the ubiquitin-proteasome pathway (Fig. 5). Atrogin-1/MAFbx and the upstream Foxo3a signaling pathway are activated in cancer cachexia^47–49^. The Foxo signaling pathway regulates muscle wasting in various cachexia-related conditions, including sepsis, cancer, and heart failure^50,51^. Here, we provide evidence that PAUF is a key factor in cancer cachexia. In vitro results showed that PAUF conditioned medium increased muscle atrophy and triggered muscle wasting pathways (Fig. 5). In addition, PAUF promoted muscle loss in a pancreatic cancer xenograft model. These results identified a direct Akt-Foxo3a-Atrogin1/MAFbx linkage involved in rPAUF-induced muscle wasting and indicated that PAUF might play a role in pancreatic cancer cachexia.

Gene ontology and KEGG analyses were performed to investigate the potential biological processes and pathways associated with PAUF (Fig. 6e). The results identified IL-8 production, the nitric oxide biosynthetic process, MyD88-dependent TLR signaling, and the TNF biosynthetic process among important biological processes related to PAUF. These findings are consistent with previous reports showing that inflammatory factors can induce muscle atrophy^52–54^. Moreover, it was suggested that PAUF might be involved in muscle atrophy-causing processes in pancreatic cancer by affecting the corresponding inflammatory signaling pathways. However, the observed protein–protein interaction networks corresponding to PAUF and the related inflammatory responses will be investigated in follow-up studies.

Pancreatic cancer biomarkers for various applications, such as early detection, prognostic prediction, and therapeutic response prediction, are urgently needed. The NLR is a prognostic biomarker in inflammatory diseases, including several types of cancer, and it is a predictor of poor survival in patients with advanced pancreatic cancer^55,56^. Recently, Basile et al. reported that early loss of skeletal muscle mass plays a negative prognostic role in advanced pancreatic cancer patients^57^. These studies are in line with our findings, which showed that plasma NLR values were significantly higher in the high-PAUF group than in the low-PAUF group. Moreover, analysis of public databases showed that PAUF expression is increased in pancreatic tumors and that increased gene expression and copy number gain of PAUF are correlated with poor overall survival (Fig. 7b, c).

The present study has several limitations, including the small number of patients and lack of sarcopenia information. In addition, PAUF was assessed at only a single time point before surgery. Multicenter studies with a large number of patients and a longitudinal study design are necessary to confirm the association between PAUF levels and cachexia in pancreatic cancer. Finally, we did not find significant differences between food intake and BMI. However, we specifically aimed to investigate PAUF as a cachectic factor associated with pancreatic cancer independent of involuntary anorexia.

In summary, our findings are critical in identifying a direct relationship between PAUF and cancer cachexia and suggest that PAUF represents a cachectic factor that could be modulated to affect the clinical outcomes of patients with pancreatic cancer. As PAUF is routinely measured at low cost in clinical practice, it could serve as a simple and convenient predictive and stratification factor to assist with clinical decision-making in pancreatic cancer.

## Supplementary information

Supplementary all
