# Supplementary material for: Pancreatic cancer induces muscle wasting by promoting the release of pancreatic adenocarcinoma upregulated factor
Source: Exp Mol Med. 2021 Mar 17;53(3):432–45. doi: 10.1038/s12276-021-00582-2 (PMC8080719; doi:10.1038/s12276-021-00582-2)
Supplement: Supplementary file 1 — Supplementary all [file 12276_2021_582_MOESM1_ESM.pdf]

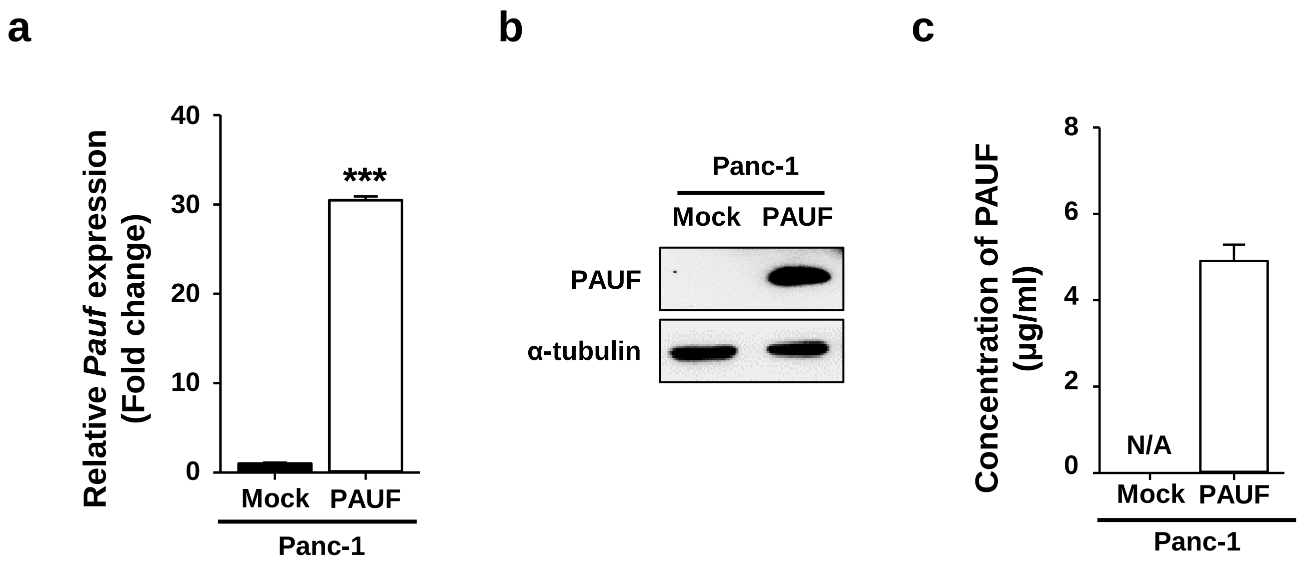

Supplementary Figure 1. Expression level of PAUF mRNA and protein in Panc-1/Mock and Panc-1/PAUF cell line.

a Quantification by RT-qPCR of *Pauf* mRNA expression levels in Panc-1/Mock and Panc-1/PAUF cell lines. mRNA expression levels were normalized with *Gapdh* mRNA. **b** Western blot analysis and **c** ELISA assay of PAUF from cell cultured medium in Panc-1/Mock and Panc-1/PAUF cell lines. The data represent the mean ± SD. *$p < 0.05$, **$p < 0.01$, and ***$p < 0.001$ vs. Panc-1/Mock cell line. N/A, not available.

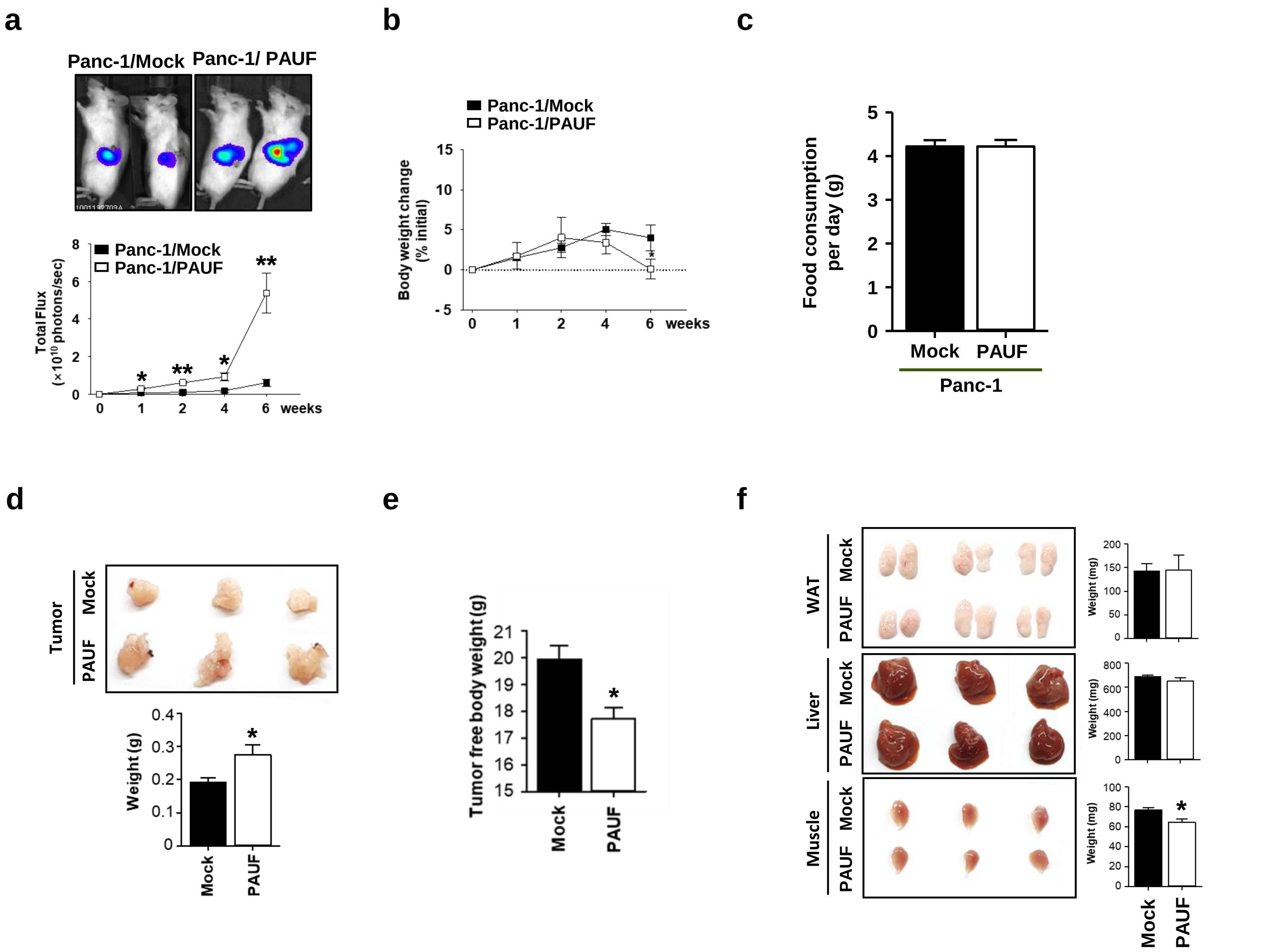

a
Panc-1/Mock
Panc-1/ PAUF
Total Flux (×10^10 photons/sec)
Panc-1/Mock
Panc-1/PAUF
weeks
b
Panc-1/Mock
Panc-1/PAUF
Body weight change (% initial)
weeks
c
Food consumption per day (g)
Mock
PAUF
Panc-1
d
Tumor
Mock
PAUF
Weight (g)
e
Tumor free body weight (g)
Mock
PAUF
f
WAT
Liver
Muscle
Mock
PAUF
Weight (mg)

Supplementary Figure 2. Association between cachexia and pancreatic cancer-derived PAUF.

Panc-1/Mock or Panc-1/PAUF cells were orthotopically injected into NSG mice. **a** Bioluminescent imaging by luciferase in Panc-1/Mock or Panc-1/PAUF cancer model and photon values of tumor mass were displayed in the graph. **b** Time-dependent changes were expressed as percent modulation of initial body weight. **c** Food consumption per day. **d** Representative tumor image and tumor weight after Panc-1/Mock cells or Panc-1/PAUF cells at 6 weeks post-injection. **e** Tumor-free body weight. **f** Representative images of white adipose tissues (WAT), liver, and tibialis anterior (TA) muscles are shown in Panc-1/Mock or Panc-1/PAUF tumor-bearing mice. Weight of WAT, liver, and TA muscle. The data represent the mean ± SD. *$p < 0.05$ and **$p < 0.01$ vs. Panc-1/Mock-injected mice.

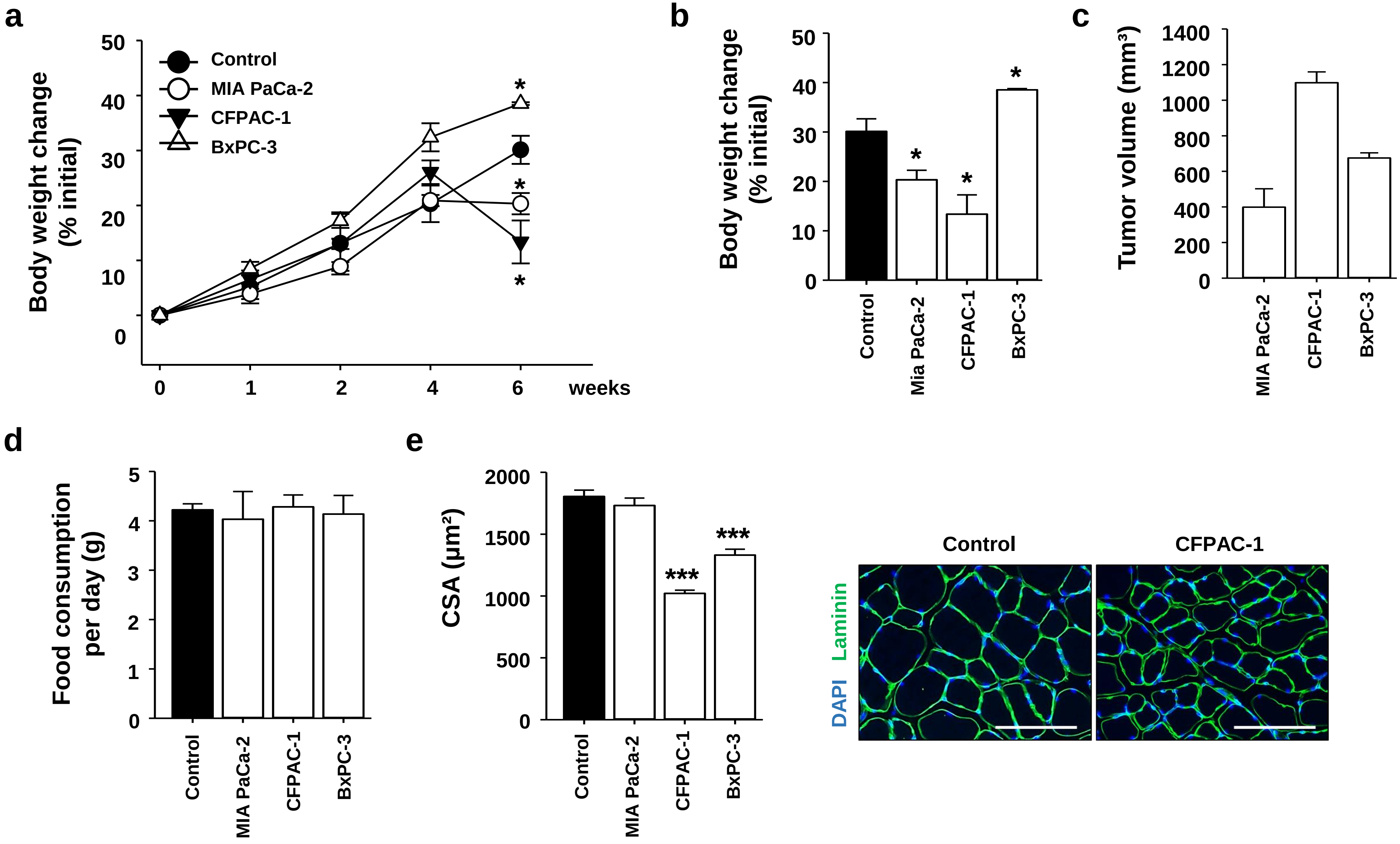

Supplementary Figure 3. PAUF-expression human pancreatic cancer cell lines induce body weight loss and muscle atrophy.

a Time-dependent changes expressed as percent modulation of initial body weight in mice injected subcutaneously with human pancreatic cancer cell lines ; MIA PaCa-2, CFPAC-1, and BxPC-3 or PBS (control). **b** Final body weight change percent compared to initial body weight and **c** tumor volume at 6 weeks post-injection. **d** Food consumption per day. **e** The graph shows the mean CSA of muscle myofibers. The data represent the mean ± SD. *$p < 0.05$, **$p < 0.01$, and ***$p < 0.001$ vs. PBS.

a b

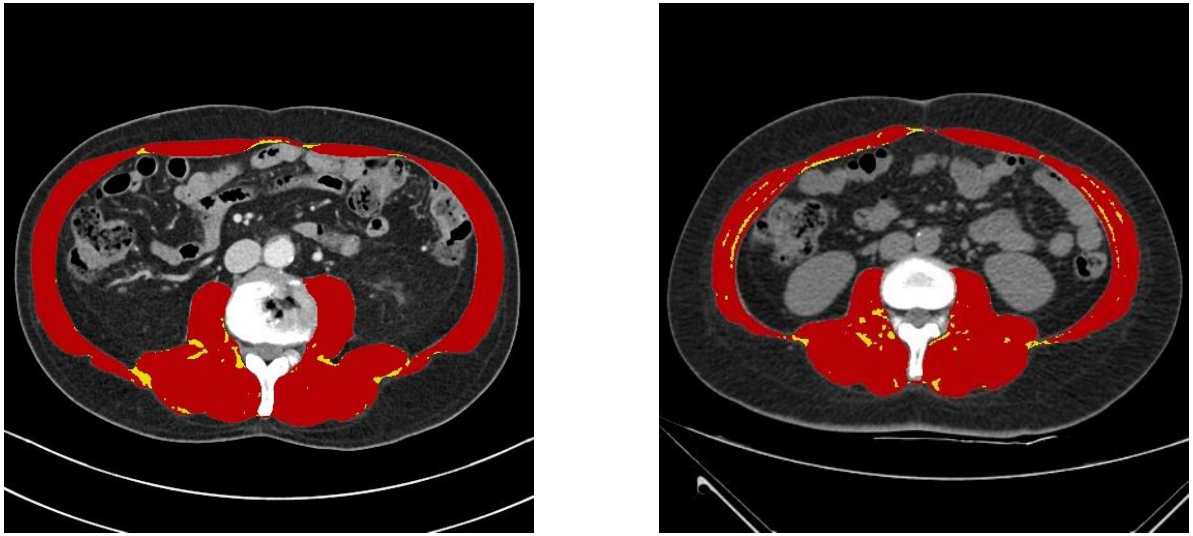

Supplementary Figure 4. Representative images of muscle and fat measurement in CT of patients.

The SMA (red colored area) and IMA (yellow colored area) at abdominal CT scan in cancer patients were segmented. **a** In 71-year old patient, CT showed large SMA and low IMA, and the plasma PAUF level was 7.33 ng/ml. **b** In 49-year old patient, CT showed small SMA and large IMA, and the plasma PAUF level was 11.63 ng/ml. (SMA; skeletal muscle area , IMA; intermuscular fat area).

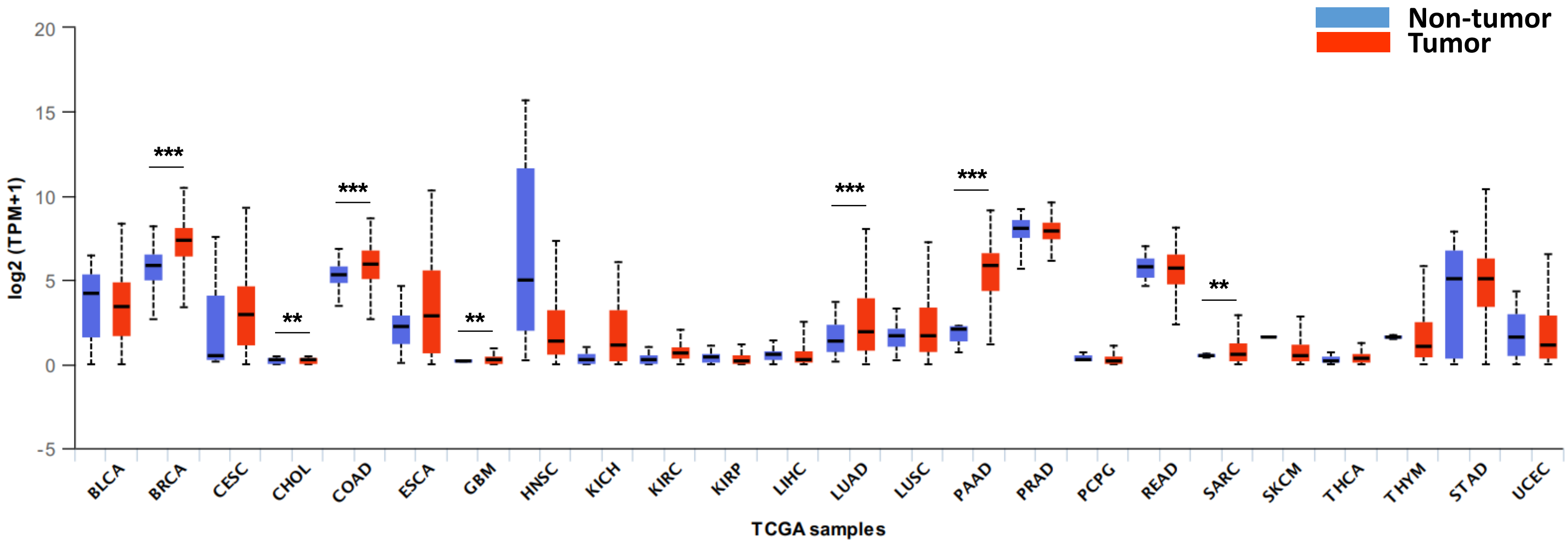

Supplementary Figure 5. Expression of PAUF across TCGA cancers (with tumor and normal samples)

BACA: Bladder urothelial carcinoma; **BRCA**: Breast invasive carcinoma; **CESC**: Cervical squamous cell carcinoma; **CHOL**: Cholangiocarcinoma; **COAD**: Colon adenocarcinoma; **ESCA**: Esophageal carcinoma; **GBM**: Glioblastoma multiform; **HNSC**: Head and neck squamous cell carcinoma; **KICH**: Kidney chromophobe; **KIRC**: Kidney renal clear cell carcinoma; **KIRP**: Kidney renal papillary cell carcinoma; **LIHC**: Liver hepatocellular carcinoma; **LUAD**: Lung adenocarcinoma; **LUSC**: Lung squamous cell carcinoma; **PAAD**: Pancreatic adenocarcinoma; **PCPG**: Prostate adenocarcinoma; **PCPG**: Pheochromocytoma and paraganglioma; **READ**: Rectal adenocarcinoma; **SARC**: Sarcoma; **SKCM**: Skin cutaneous melanoma; **THCA**: Thyroid carcinoma; **THYM**: Thymoma; **STAD**: Stomach adenocarcinoma; **UCEC**: Uterine corpus endometrial carcinoma

Supplementary Table 1. Hematological characteristics of pancreatic cancer patients

Variable	**ALL**	**Low PAUF** (PAUF < 10.2ng/ml)	**Hihg PAUF** (PAUF ≥ 10.2ng/ml)	*p*-value
**Hb (g/dL)**	12.9 (±1.4)	12.9 (±1.7)	12.9 (±1.2)	
**Hct (%)**	38.8 (±4.3)	38.6 (±5)	39.1 (±3.7)	
**Platelet**	242.1 (±80.8)	249.5 (±81.5)	233 (±83.8)	
**monocyte (%)**	7.3 (±1.9)	6.6 (±1.6)	8.2 (1.9)	
**PT (sec)**	11.8 (±1.2)	11.4 (±0.7)	12.4 (1.5)	
**aPTT (sec)**	27.7 (±3.3)	27 (±2.8)	28.6 (±3.9)	
**AST (IU/L)**	46.7 (±75.9)	26.4 (±18.4)	71.4 (±109.5)	n.s[a]
**ALT (IU/L)**	54.9 (101.5)	24 (±18.9)	92.7 (±145.2)	
**ALP (IU/L)**	115 (±90.2)	101.1 (±41.1)	132 (±129)	
**Tbil (mg/dL)**	0.8 (±0.6)	0.7 (±0.5)	1 (±0.7)	
**BUN (mg/dL)**	14.6 (±5.9)	14.2 (±6.1)	15.1 (±5.9)	
**Creatinine (mg/dL)**	0.8 (±0.2)	0.8 (±0.2)	0.8 (±0.2)	
**CA19-9 (U/mL)**	332.2 (±396.4)	312.2 (±384)	356.5 (±433.3)	

Values are means ± standard deviations.
[a]Mann–Whitney U-test.
Hb; Hemoglobin, Hct; Hematocrit, PT; Prothrombin Time, aPTT; activated Partial Thromboplastin Time, AST; Aspartate Aminotransferase, ALT; alanine aminotransferse, ALP; Alkaline Phosphatase, Tbil; Total Bilirubin, BUN; Blood Urea Nitrogen.
